# Anesthetic effects of the ketamine and midazolam association by intranasal or intramuscular route in domestic chickens: prospective, blinded, randomized and crossover study

**DOI:** 10.29374/2527-2179.bjvm005923

**Published:** 2024-07-04

**Authors:** Fernanda Meirelles Adão, Isabella Danon Martins, Álvaro Alberto Moura Sá dos Passos, Renata Fernandes Ferreira de Moraes, Daniel de Almeida Balthazar, Eduardo Butturini de Carvalho

**Affiliations:** 1 Veterinarian, Autonomous, Rio de Janeiro, RJ, Brazil.; 2 Undergraduate in verterinary medicine, Universidade de Vassouras, Vassouras, RJ, Brazil.; 3 Veterinarian, MSc. Universidade de Vassouras, Vassouras, RJ, Brazil.; 4 Veterinarian, DSc., Universidade de Vassouras, Vassouras, RJ, Brazil.; 5 Veterinarian, DSc, Departamento de Medicina e Cirurgia Veterinária, Instituto de Veterinária, Universidade Federal Rural do Rio de Janeiro, Seropédica, RJ, Brazil

**Keywords:** conscious sedation, Gallus gallus, ketamine, benzodiazepine, birds, sedação consciente, Gallus gallus, ketamina, benzodiazepina, aves

## Abstract

This prospective, blinded, randomized crossover study aimed to assess the anesthetic effects of the combination of 30 mg/kg ketamine and 2 mg/kg midazolam via intranasal (IN) or intramuscular (IM) routes in twelve domestic chickens. Physiological parameters (respiratory rate – RR, heart rate – HR, and cloacal temperature –Tºcloacal) were monitored throughout the experiment, along with recovery time and sedation level (S0: awake, no recumbency, responsive to stimuli; S1: blinking eyes, recumbency, relaxed, response to stimulus, mild movement; S2: open eyes, recumbency, relaxed, mild response to stimuli; S3: closed eyes, recumbency, relaxed, no movement). In the IM group, all birds reached S3, while in IN 5/12 reached S3, 4/12 reached at most S1, and 1/12 at most S2. IM administration showed higher sedation at 5, 10, 15, 20, 30, 35, 40, and 45 minutes (p<0.05). IN administration exhibited a shorter total recovery time (26.3±21.4 min vs. 92.9±33.4 min; p<0.001). No time, group, or time-group interaction effects were observed in HR and cloacal Tº, with a trend to a decrease in RR both groups (p<0.001). Increased incidences of vocalization and agitation was observed via IM (4/12 vs. 0/12; p=0.028), with no difference in salivation. Despite faster recovery with less agitation and vocalization, the ketamine and midazolam combination via IN provided less consistent sedation compared to the IM route in chickens.

## Resumo

Este estudo *crossover* randomizado objetivou avaliar os efeitos anestésicos da associação de 30 mg/kg de cetamina e 2 mg/kg de midazolam via intranasal (IN) ou intramuscular (IM) em doze galinhas. Além dos parâmetros fisiológicos (frequência respiratória – FR e cardíaca – FC e temperatura cloacal – Tºcloacal), registrou-se o tempo de recuperação e o grau de sedação ao longo do experimento (S0: acordada, sem decúbito, responsiva a estímulos; S1: olhos piscando, decúbito, relaxada, resposta a estímulo, movimentação leve; S2: olhos abertos, decúbito, relaxada, resposta leve a estímulos; S3: olhos fechados, decúbito, relaxada, sem movimentação. Pela via IM, todas as aves atingiram o grau S3, enquanto pela via IN 5/12 alcançaram S3, 4/12 atingiram no máximo S1 e 1/12 no máximo S2. A via IM apresentou maior sedação em 5, 10, 15, 20, 30, 35, 40 e 45 min (p<0,05). A via IN apresentou menor tempo total até recuperação (26,3±21,4 min *vs*. 92,9±33,4 min; p<0,001). Não foram observados efeitos de tempo, grupo e interação tempo-grupo na FC e na Tºcloacal com uma tendência de queda da FR nos dois grupos (p<0,001). Observou-se maior incidência de vocalização e agitação pela via IM (4/12 *vs.* 0/12; p=0,028), não havendo diferença para sialorreia. Apesar da recuperação mais rápida e com menos agitação e vocalização, a associação cetamina e midazolam via IN levou a uma sedação menos consistente que a via IM em galinhas.

## Introduction

Physical restraint of birds can be a challenging procedure, with the risk of injury to both the animal and the handler (Cabanac & Aizawa, 2000). The high level of stress experienced by the bird during restraint may be associated with complications such as hyperthermia, trauma, tachypnea, syncope, and even death in critical patients (Mans, 2014; Schäffer et al., 2016). Therefore, chemical restraint may be an option to reduce stress and the risk of injuries to both the bird and the handler during restraint, enabling the performance of diagnostic and therapeutic procedures (Mans, 2014; Conner et al., 2022). Drug selection and the respective doses, as well as the routes of administration should take into consideration individual factors, such as species, behavior, and comorbidities, as well as factors related to sedatives and anesthetics, including their pharmacokinetic and pharmacodynamic characteristics.

Among the possible routes of administration, the intramuscular (IM) route through the pectoral muscle is widely used in routine procedures. However, this route may induce pain, stress, hemorrhages, and even alterations in biochemical tests (Mans, 2014).The intranasal (IN) route has shown promising results, especially for inducing minimal stress and less pain during administration, requiring a short duration of physical restraint (Vesal & Zare, 2006; Sadegh, 2013; Hornak et al., 2015a; Raisi et al., 2016; Schäffer et al., 2016; Altundag et al., 2021; Conner et al., 2022). Studies have demonstrated that benzodiazepines (Vesal & Zare, 2006; Sadegh, 2013; Hornak et al., 2015a; Raisi et al., 2016; Schäffer et al., 2016; Altundag et al., 2021; Vajdi & Alian Samakkhah, 2023) or the combination of these drugs with ketamine via the intranasal (IN) route may be associated with effective sedation in different bird species. However, specific protocols for IN sedation or anesthesia in birds, especially involving the combination of midazolam with ketamine, are not yet well-established. The aim of this study was to compare the clinical effects of the combination of ketamine with midazolam via the intranasal (IN) route as opposed to the intramuscular (IM) route in domestic chickens.

## Material and methods

This prospective, randomized, blinded, and crossover study was approved by the animal ethics committee (Universidade de Vassouras) under protocol 012/2018. Twelve healthy adult domestic chickens (*Gallus gallus domesticus*) were included. Their health status was confirmed through a physical examination conducted by a veterinarian with over 10 years of experience in avian medicine. Chickens were housed in a communal aviary with access to water and specific *ad libitum* feed.

The animals were randomly assigned to two groups: intramuscular (IM) and intranasal (IN). Following a crossover design, each animal switched groups after a 15-day interval from the first phase of the experiment.

**Figure 1:**
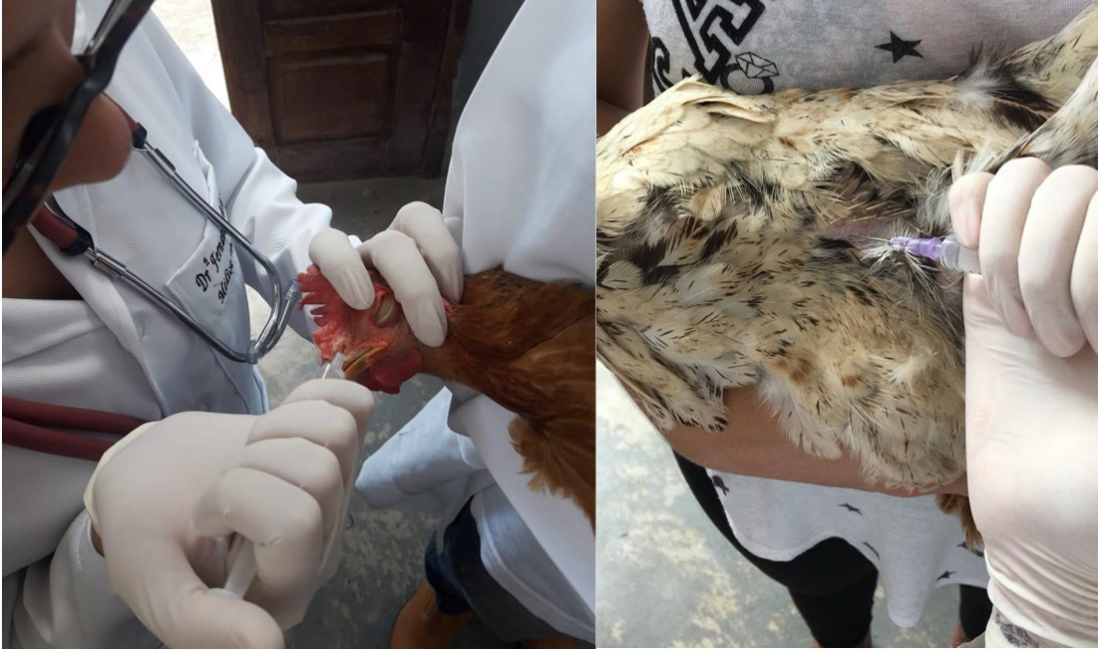
Left – intranasal (IN) administration into the left nostril of a domestic chicken. Right – intramuscular (IM) administration of the anesthetic solution (ketamine and midazolam). Source: personal archive, 2018.

On experimental days, the chickens underwent a 4-hour fasting period for food and water, followed by weighing. The anesthetic protocol involved the IM or IN administration of a combination of 30 mg/kg ketamine (Cetamine® 100mg/ml, Vetnil – Louveira, SP) and 2 mg/kg midazolam (Dormire® 5mg/ml, Cristália – São Paulo, SP). Animals were physically restrained by the same researcher in dorsal recumbency, and the anesthetics were slowly administered either into one nostril (IN) or into the pectoral muscle (IM), as depicted in Figure 1.

After administration of the solution, animals were transferred to individual, quiet, and low-light stalls, positioned in right lateral recumbency for sedation assessment – conducted by a blinded veterinarian unaware of the bird's group and using the scale shown in Chart 1. In addition to sedation, the following parameters were measured and recorded every 5 minutes: heart rate (HR) by auscultation in beats per minute (bpm), respiratory rate (RR) in movements per minute (mpm) by observing pectoral muscle movement and auscultation, cloacal temperature (TºC) by digital thermometry, and time to recovery (ranging from the start of anesthetic administration until the return to S0). The incidences of vocalization, agitation, and drooling during recovery were also recorded. All animals were monitored until full recovery and then returned to their aviaries.

**Chart 1:** Chart describing the bird sedation scale.

**Bird sedation level**	
**Level**	**Characteristics**
0	Awake, without recumbency, responsive to stimuli.
1	Blinking eyes, recumbency, relaxed, responsive to stimulus, slight movement.
2	Open eyes, recumbency, relaxed, mild response to stimuli.
3	Closed eyes, recumbency, relaxed, no movement.

Data were analyzed using Jamovi 2.3.28 software (graphs created in GraphPad Prism v9.0), tested for normality using the Shapiro-Wilk test, homogeneity of variances with Levene's test, and sphericity with Mauchly's test. Data are presented as mean ± standard deviation when appropriate for normal distribution, or median (range) when not suitable for normal distribution. Sedation and recovery quality scales over time were compared using the Kruskal-Wallis test with Dwass-Steel-Critchlow-Fligner *post-hoc*. Recovery time was compared using the Student's t-test, and physiological parameters (HR, RR, and TºC up to 60 minutes) were compared using repeated measures ANOVA with the Greenhouse-Geisser correction and the parameter at T0 as a covariate. The comparison of the incidence of adverse effects was performed using the Chi-square test. All tests had a statistical significance level of 0.05.

## Results

**Figure 2:**
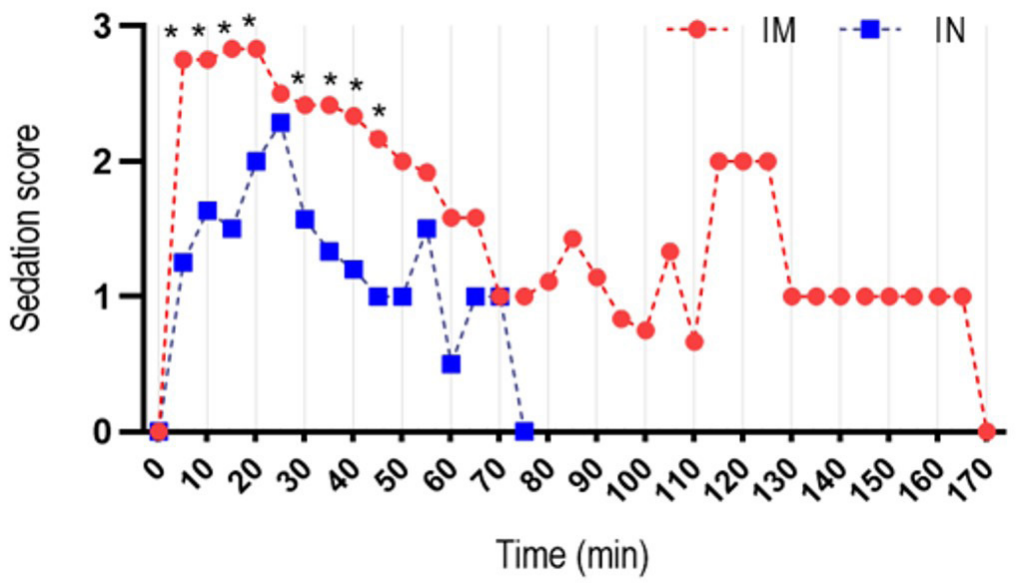
Mean sedation scores over time in minutes (min) for birds sedated with the combination of 30 mg/kg ketamine and 2 mg/kg midazolam via intramuscular (IM) or intranasal (IN) routes. The times marked with * showed statistical differences between the groups.

The average weight of the birds was 1.800 ± 0.533 kg, and there were no fatalities during the study. In the IM group, all animals reached the maximum sedation level (S3), while in the IN group, 1/12 (8.3%) did not reach even S1, remaining without signs of sedation. Additionally, 4/12 (33.3%) reached a maximum of S1, 1/12 (8.3%) reached a maximum of S2, and 5/12 (41.7%) reached S3. The sedation scale differed significantly between the IM and IN groups at 5 min (p=0.003, ε^2^=0.380), 10 min (p=0.013, ε^2^=0.278), 15 min (p=0.002, ε^2^=0.476), 20 min (p=0.028, ε^2^=0.254), 30 min (p=0.025, ε^2^=0.278), 35 min (p=0.014, ε^2^=0.356), 40 min (p=0.026, ε^2^=0.308), and 45 min (p=0.029, ε^2^=0.319). Figure 2 presents the mean sedation scores for the IM and IN groups over time.

**Figure 3:**
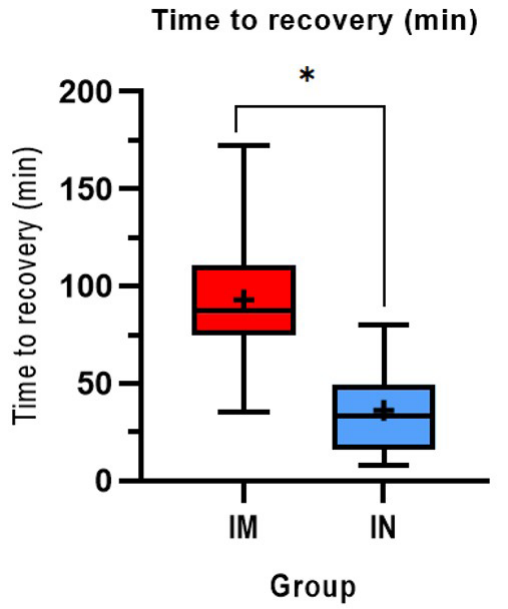
Boxplot of recovery times from sedation (return to S0) for birds sedated with the combination of 30 mg/kg ketamine and 2 mg/kg midazolam via intramuscular (IM) or intranasal (IN) routes. * - p<0.001. The symbol '+' represents group mean.

Mean time to complete recovery (return to S0) is depicted in Figure 3, being 92.9±33.4 min in the IM group and 26.3±21.4 min in the IN group, with a statistically significant difference (p<0.001). The minimum and maximum times until recovery were, respectively, 35 and 172 min in IM and 8 and 80 min in IN.

**Figure 4:**
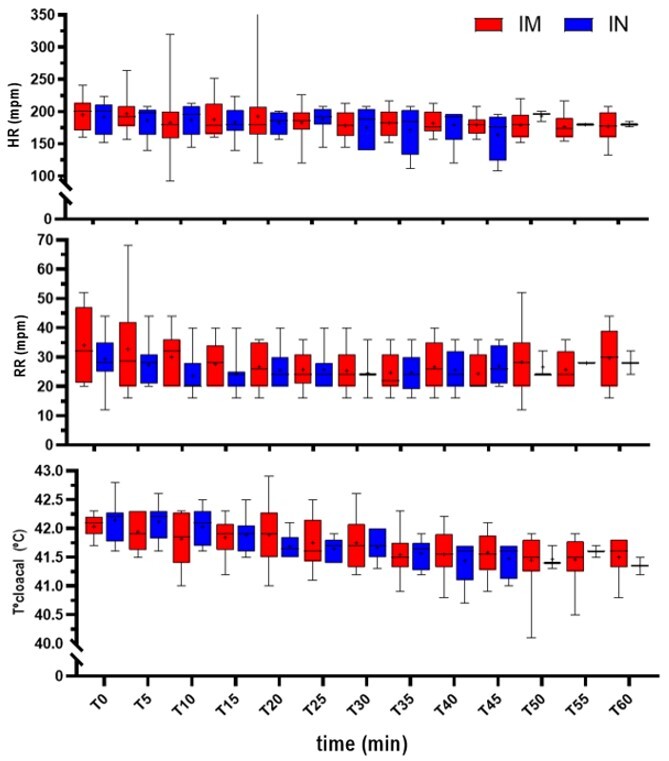
Boxplot of heart rate (HR), respiratory rate (RR), and cloacal temperature (Tº) during sedation of birds with the combination of 30 mg/kg ketamine and 2 mg/kg midazolam via intramuscular (IM) or intranasal (IN)routes. The symbol '+' represents group mean. Bpm – beats per minute; mpm – movements per minute.

In the first 60 minutes of the experiment, there was no effect on heart rate (HR) of: time (p=0.476), route of administration (p=0.796), or the interaction between time and route of administration (p=0.715). During the same period, there was a trend of decreasing respiratory rate (RR) over time (p<0.001; η^2^=0.062), with no effect of the interaction between time and route (p=0.972) or the route of administration (p=0.795). Regarding cloacal temperature (Tºcloacal) during the same period, there was no effect of time (p=0.541), the interaction between time and route (p=0.802), or the route of administration (p=0.835). Figure 4 presents the physiological parameters (HR, RR, and Tºcloacal) during the first 60 minutes of the experiment in both groups.

The IM route was associated with a higher incidence of vocalization and agitation during recovery (4/12 vs. 0/12, p=0.028), with no difference in drooling (2/12 vs. 1/12, p=0.537).

## Discussion

The administration of the combination of 30 mg/kg ketamine with 2 mg/kg midazolam via IM led to more consistent sedation in chickens compared to the IN route. In this group, one animal did not reach even the lowest sedation level, and only 5 out of 12 reached the maximum level, while with the IM route, all animals reached the maximum sedation level. This result may be due to the greater difficulty in ensuring the absorption of the entire instilled content into the nostrils of the birds compared to IM administration. Although rarely reported in the literature (Schäffer et al., 2016), the sneezing reflex after the instillation of sedatives can lead to the loss of part of the administered content. A study in *Melopsittacus undulatus* (Sadegh, 2013) demonstrated that IN midazolam was able to provide satisfactory sedation. However, despite not using ketamine in combination, the dose of midazolam used was 13 mg/kg, much higher than the dose adopted in the current research.

Observing the mean sedation scales - Figure 2, the peak of sedation occurred in the first 5 minutes when the IM route was used, sustaining an effect up to S2 for approximately 50 minutes. In the case of the IN route, the highest values occurred only at 20-25 minutes. This result contrasts with those obtained in three studies: the first in *Melopsittacus undulatus* with intranasal midazolam (1.3±0.44 minutes) (Sadegh, 2013), the second in *Pyrrhocorax pyrrhocorax* (Raisi et al., 2016), 4±2.9 minutes, and the last in parakeets (Vesal & Eskandari, 2006), where the average onset of sedation occurred in just 2.2±0.4 minutes. One hypothesis for this difference lies in the doses of each drug used: 13 mg/kg (Sadegh, 2013), 8 mg/kg (Raisi et al., 2016), and 3.65 mg/kg of midazolam (Vesal & Eskandari, 2006), and 40-50 mg/kg of ketamine, in the latter case, almost 70 to 80% higher than the doses used in the present study.

Another significant difference between the routes of administration was the recovery time. Through the IN route, the longest sedation lasted 80 minutes, but the mean was only 26 minutes, a time similar to that observed with IN administration of midazolam (5mg/kg) in pigeons (Hornak et al., 2015b). Through the IM route, the mean was 93 minutes, with one bird taking 170 minutes to recover. In a study in parakeets (*Psittacula krameri*), the same drug combination promoted sedation for 210.8±17.5 minutes, considerably longer than the values obtained herein (Vesal & Eskandari, 2006). A study with IN midazolam only in *Melopsittacus undulatus* provided 71.6±8.9 minutes of sedation (Sadegh, 2013). However, the longer duration of the sedative effect may have been due to the higher doses of each drug used, as presented in the previous paragraph.

No significant changes were observed in physiological parameters in the current study, similar to the findings during sedation in *Myiopsitta monachus* with midazolam (2 mg/kg) and butorphanol via IN (Conner et al., 2022), and in pigeons sedated with IN midazolam (Hornak et al., 2015b). Only a slight trend to a reduction in RR (mean effect- η^2^=0.062) was observed, independent of the route of administration, likely more due to the reduction in stress caused by sedation than by the depressant properties of the drugs, an effect also observed in a study with parrots (*Amazona ventralis*) sedated only with midazolam via IN (2 mg/kg) (Mans et al., 2012). Cloacal temperature does not seem to have been affected by sedation. Parrots in the above-mentioned study showed a tendency to increase in temperature during the experiment; however, the animals were kept under physical restraint during the experiment and received only midazolam, suggesting that the temperature increase may have occurred due to restraint under a lower level of sedation. On the other hand, pigeons sedated with midazolam showed a slight decrease in cloacal temperature, still remaining within physiological values (Hornak et al., 2015b)

Both routes of drug administration proved to be quite safe, not causing deaths or major complications. Sialorrhea occurred in only a few animals (2/12 via IM and 1/12 via IN), and vocalization and agitation during recovery were observed only via IM in 33% of the animals. Sedation with the same protocol in *Pyrrhocorax pyrrhocorax* demonstrated low-quality recovery in only 1/7 animals (Raisi et al., 2016).

## Conclusion

The combination of 30 mg/kg ketamine with 2 mg/kg midazolam via IN produced less consistent sedation than the IM route, although half of the birds showed at least: spontaneous recumbency, generalized relaxation, and a mild response to stimuli. This result, combined with the rapid recovery (average of 26 min), suggests sufficient sedation for the performance of small procedures, with stability in heart and respiratory rates and temperature.
